# Care of the Mouth in Relation to Prophylaxis of the Digestive Tract

**Published:** 1897-07

**Authors:** G. V. E. Brown


THE
AMERICAN JOURNAL
—OF—
DENTAL SCIENCE.

VOL. XXXI. BALTIMORE, JULY, 1897. NO. 3.

ARTICLE I.

### CARE OF THE MOUTH IN RELATION TO PROPHYLAXIS OF THE DIGESTIVE TRACT.

BY G. V. E. BROWN.

A discussion of the subject under consideration necessarily concerns the two principal offices of the oral cavity in the process of alimentation and pathological conditions originating in this region, viz: Mastication, insalivation, reflex nervous disturbances, micro-organisms.

The proper trituration of articles of food depends upon the perfect occlusion of the morsal surfaces of the teeth, and is absorbed by malformation of the jaws, loss of the teeth, or destruction of their crowns from caries or other causes; by artificial dentures and bad habits of mastication.

For prevention of trouble from imperfections of the teeth the physician must naturally have recourse to the services of a dentist, but inquiry into the habits of mastication, and in this regard no greater assistance can be given than an effort to make the environments during the hours for eating such as will have a quieting mental influence and assist deliberation in all of its processes.

Upon the action of the saliva is dependent digestion of the starches and their canversion into sugar, the process continuing in the stomach after swallowing, and if this action upon the carbohydrates be insufficient, such matter becomes a source of irritation and medium for the ferment-producing bacteria, or, if passed into the intestines in an undigested state, will overtax the digestive action there and cause intestinal disturbances.

Here again mental preoccupation or mental distress must be so far as possible allayed, as upon an active consciousness of the gustatory function is dependent, in a large measure, the flow of the saliva from its glandular sources. Again, also, we find this dependent upon the effect of masticatory effect, as the activity of the parotid is much stimulated by the pressure of the ramus of the inferior maxillary, hence undue haste must necessarily result in the swallowing of a bolus of food improperly mixed with saliva. The wearers of ill-fitting artificial dentures, by reason of the dislodgement suffered in opening the jaws wide, or the dropping down on one side during an effort to crush or masticate upon the opposite side of the mouth, acquire the habit of a sliding movement and do not open and shut the jaws freely. They thus become frequent sufferers from digestive disturbances, hence the erroneous theory (still extant, I believe, among certain homœopaths) concerning the poisonous influence of mercury set free from the vulcanization of the red-rubber plates.

Reflex action from inflammations of tooth-pulps, pericementum and gums, while widespread in its general manifestations upon other parts, has comparatively infrequent direct influence upon the digestive tract, yet there are well known ill effects accompanying the irritation of first dentition, not, as frequently assumed, by the inflamed condition of the gums, but by the pressure of the still open end of the root in its imperfect state of development upon the pulp of the tooth, and its nerve connection caused by the tough overlying tissue resisting nature's efforts to push the crown through as growth takes place. In like manner we sometimes find difficulty in the eruption of the third molars (so-called wisdom teeth). The lance can of course largely prevent such disturbances.

The necessity and value of sterilization of the secretions of the mouth, as a prophylactic measure, with references to diseases of the stomach and intestines, depend upon the nature of the micro-organisms found there, and whether these can withstand the action of the gastric juices long enough to become excitants of pathological conditions, or pass through into the intestines with sufficient vitality to reproduce themselves and there also contlnue to be breeders of disease.

These points have been most carefully determined by Dr. Miller, of Berlin, and Dr. Fenton B. Turck, of Chicago, to whom I am indebted for valuable literature upon the subject.

Dr. Miller was able, in an uncleanly mouth, to estimate by culture methods 1,149,000,000 cultivable bacteria. He isolated twenty-five different kinds, and of these twelve were again found in the fœces, eight in the stomach. He demonstrated that there were five forms of gas-producing bacteria. As a result of his series of experiments he gives the following conclusions:

1. The microbes which are swallowed at the beginning of a meal do not pass into a stomach filled with gastric juice, but into an empty stomach having a neutral or alkaline reaction when free from hydrochloric acid, which does not appear in detectable quantities until after the lapse of one and one-half hours.

2. The micro-organisms are often imbedded in solid particles of food, thus escaping for a while the action of the juices.

3. Liquid substances do not remain long in the stomach, but soon pass into the duodenum and carry with them the bacteria before any considerable amount of gastric juice has been secreted.

Turck says: "It has been observed that pathogenic micro-organisms may be swallowed and still no infection of the stomach be apparent, but let some errors of diet, the abuse of alcohol, irregularities of living take place, then the mucous membrane forms a fertile soil for the development of the micro-organisms." He calls attention, further, to the fact that the bacillus coli communis developing in the lumen produces no marked change, but when developed on the walls it lowers the vitality of the cells and paves the way for more active pyogenic organisms, or if imprisoned in the tissues it at once becomes a pyogenic organism.

The following case, described by Turck, I quote, as it illustrates the value of an accurate demonstration of existing bacteriological conditions in harmony with correct diagnosis in actual practice, by which the hitherto speculative status of this subject is at once removed.

"Cultures were made from the mouth, in which were found large numbers of bacillus lacticus; germs of putrefaction, as well as fermentation and staphylococci. Pyogensaures were also found in an abscess in the mouth. Cultures were made from the stomach also and the bacillus lacticus was found, groups of thread-form bacilli and staphylococci were also found. It was a severe case of rapid gastric inflammatory process with beginning of atraphy. Disinfection of the mouth and placing the teeth in good order were the first indications in the case. A similar disinfection of the stomach with soap and water. followed by lysol, was instituted. An improvement in the case was manifested at once."

In other cases, cited by the same author, complete cures were effected simply by treating the mouth and showed the same corroborative bacteriological testimony.

Disinfections in the oral secretions must include such operative measures as may be necessary for the removal of exciting causes—filling, treatment, or extraction of carious or diseased teeth and roots, diseased bone; cleansing of pockets and deposits about the necks of teeth. The regulation of the position of the teeth in cases presenting malformations, removal of artificial dentures with, if possible, a metallic base to give an increased healthfulness of the covered mucous membrane, by reason of better conducting properties; if not removal, at least the correction of mal-constructed bridge-work and crowns. All this of course pertains to the dentist's portion, and concerns the physician only in that if done according to his personal direction, as to the intended result, the dentist's services will be usually found to be much more effective, but the use of an antiseptic mouth wash can do wonders of itself by repeated application and is easily within the province of every practitioner of medicine.

The difficulties encountered in the sterilizing of the buccal secretions are: 1. By reason of dilution with saliva, any drugs sufficiently powerful to destroy germs are rendered almost, if not entirely, inert. 2. The short time of exposure during the process of rinsing the mouth is insufficient for those of slow action, especially when particles of solid food are impacted in the teeth. 3. Danger of injurious effects on the tooth structure. 4. Unpleasant tastes are objectionable.

With these essential facts in view, the preparation of a suitable mouthwash prescription becomes a simple matter, and further reference to extensive literature on the subject is unnecessary.

Soap, applied with a brush, is one of the best cleaners, by reason of its alkaline properties dissolving the mucous. In my own practice I have patients hold in their mouths, from two to five minutes, a solution of 1-1,000 bichloride of mercury and hydrogen dioxide, equal parts. This insures some degree of safety to both patient and operator, and upon dismissal give a prescription as follows:

℞ Listerine.
Glycerine, aa, ℥ iii
Carbolic acid, ʒ ii

M. Sig.: Dilute one-half teaspoonful in one-third glass of water.

Hold in the mouth and continue the cleansing process carefully by the watch for at least two minutes. I have found this to be effective, agreeable to most persons, and valuable by reason of its simplicity, for patients can safely vary the strength in using it from time to time, as the condition may seem to require.

During active suppuration it is sometimes necessary to urge the use of such a mouthwash every hour for a few days, but two or three times in twenty-four hours is ordinarily sufficient.

In a paper read before the last meeting of the American Medical Association I stated that I believed a part of all treatment in infectious diseases, febrile disorders, and diseases of the stomach and intestines should be a thorough disinfection of the mouth; that 95 per cent of, if not indeed all, patients would have present in the mouth some condition favorable to pathogenic organisms.

My own experience is filled with illustrations of cases, including a variety of gastric and intestinal diseases, relieved and cured by simple removal of exciting causes and disinfection on the oral cavity. What is true of my own experience is also true of all others who have given the matter active attention.

In conclusion, the question naturally arises: How much consideration of mouth diseases is possible for the general practitioner in the practical application to general practice, without expert knowledge and without being able to get the assistance of a dentist in more than a small proportion of his patients? As an answer I submit the following: In making the usual examination of the tongue, while noting its indications, look about the mouth and note:

1. The general condition, cleanly or otherwise; lesions of the mucous membrane surfaces; teeth missing, or with carious crowns, old roots, bridge-work, gold or other artificial crowns.

2. Examine surfaces of the gums for fistulous openings of alveolar abscesses, or for any sign of discharge from diseased bone.

3. If artificial dentures be worn, notice if firmly in place by making patient open and close the jaws, also the state of uncleanliness.

4. See if there be deposits of tartar and other matter about the necks of the teeth; make pressure on the gums to test for discharge of pus.

5. In children's mouths look for the eruption of teeth expected at that age.

6. With symptoms of chronic nasal catarrh, having unilateral appearance, be on guard for trouble with the maxillary sinus and examine carefully the region of the upper jaw, from cuspid to second molar, for indications of a dead root or tooth, or history of one having been extracted which might have been an exciting cause.

7. Do not be deceived, even though the mouth be cleanly in appearance and filled with shining evidence of the dentist's handiwork. Examine each tooth in as strong light as possible, compare color with that of its neighbors in the arch, and if one be slightly darker than the rest it may indicate a dead pulp, and if the history of treatment does not account for its color, quite probably the pulp-chamber of such a tooth is filled with putrescent matter from which might be ignited an infection at any favorable opportunlty.

8. Percussion with some light metallic instrument will often reveal, by pain or difference in sound, an affected tooth. Thus, by simple methods of observation, nearly all the pathological conditions of the mouth can be readily diagnosed, and the advisabilty of the disinfection at least determined while engaged in the daily round of visits.

Whenever the internal administration of antiseptics is indicated, is it not reasonable to assume, in view of the testimony, that the beginning of the digestive tract should be cared for?

As a last word, and even at the risk of irrelevancy, I

wish particularly to call attention to the consideration of the important bearing of this subject in cases of appendicitis, and, without entering into a discussion of this subject, to suggest that with the powers of resistance impaired in the vicinity of the seat of the inflammation, constant passage of pyogenic organisms must be a dangerous and inviting cause of infection, at least sufficiently so to warrant as rational the effort to prevent their introduction from the mouth. Especially is this true in cases that show a tendency to recur from time to time without operation, and doubtless many an otherwise aseptic operation may have been rendered useless by disregard of this precaution.—*Exchange.*

